# Quorum-sensing molecules: Sampling, identification and characterization of *N*-acyl-homoserine lactone in *Vibrio* sp

**DOI:** 10.1016/j.sjbs.2021.12.062

**Published:** 2022-01-04

**Authors:** Noha Laj, Muhammed Elayadeth-Meethal, V. Aldous J. Huxley, Raishy R. Hussain, Mohamed Saheer Kuruniyan, Punnoth Poonkuzhi Naseef

**Affiliations:** a A J College of Science and Technology, Trivandrum 695317, Kerala, India; b Regional Research and Training centre, Kakkur, Department of Animal Breeding and Genetics, College of Veterinary and Animal Sciences, Kerala Veterinary and Animal Sciences University, Pookode, Wayanad 673576, Kerala, India; c Thiru. Vi. Ka Govt. Arts College, Thiruvarur 610003, Tamil Nadu, India; d Department of Dental Technology, College of Applied Medical Sciences, King Khalid University, Abha 61421, Saudi Arabia; e Department of Pharmaceutics, Moulana College of Pharmacy, Perinthalmanna, Kerala 679321, India

**Keywords:** Homoserine lactones, Quorum sensing, Signaling molecules, Gram-negative bacteria, Autoinducer, *Vibrio sp*

## Abstract

Quorum sensing (QS) is a mechanism by which gram-negative bacteria regulate their gene expression by making use of cell density. QS is triggered by a small molecule known as an autoinducer. Typically, gram-negative bacteria such as *Vibrio* produce signaling molecules called acyl homoserine lactones (AHLs). However, their levels are very low, making them difficult to detect. We used thin layer chromatography (TLC) to examine AHLs in different *Vibrio* species, such as *Vibrio alginolyticus*, *Vibrio parahemolyticus*, and *Vibrio cholerae*, against a standard- *Chromobacterium violaceum*. Further, AHLs were characterised by high-performance liquid chromatography (HPLC) and gas chromatography-mass spectrometry (GC–MS). C4-HSL (N- butanoyl- L- homoserine lactone), C6-HSL (N- hexanoyl- L- homoserine lactone), 3-oxo-C8-HSL (N-(3-Oxooctanoyl)-DL-homoserine lactone), C8-HSL (N- octanoyl- L- homoserine lactone), C_1_10-HSL (N- decanoyl- L- homoserine lactone), C12-HSL (N- dodecanoyl- L- homoserine lactone) and C14-HSL (N- tetradecanoyl- L- homoserine lactone) were identified from *Vibrio*. These results may provide a basis for blocking the AHL molecules of *Vibrio*, thereby reducing their pathogenicity and eliminating the need for antimicrobials.

## 1 Introduction

Quorum sensing coordinates behaviour at the population level in bacteria. Here, the stimulus refers to bacterial density. The signal is transmitted through molecules released by the cells. Bacteria secrete acyl homoserine lactones (AHL), furanosyl borate diesters, and oligopeptides to communicate and sense density (Vesty et al., 2020, Mok et al., 2003); Nealson et al.,1970 reported a link between *Vibrio fischeri* and bioluminescence, demonstrating the phenomenon of quorum sensing Nealson et al., 1970. As per Nealson, when the density of *Vibrio fischeri* cells reaches a certain level, autoinducers are released into the aquatic environment, causing fluorescence. Gram-negative bacteria produce AHLs by using the Lux I family of enzymes Dong et al., 2017. S-adenosylmethioine (SAM) is acylated by acyl-ACP or acyl carrier protein. If AHL concentration reaches a particular threshold level, LuxR binds it, allowing it to perform various functions like bioluminescence, virulence, motility, pigment production, and antibiotic synthesis (Sholpan et al., 2021, Xue et al., 2021, Passos da Silva et al., 2017, Mukherjee and Bassler, 2019, Kamareddine et al., 2018). It has been found that AHL molecules influence about 37 genera of gram-negative bacteria (Zhao et al., 2021).

A variety of small atoms called auto inducers (AIs) are used in *Vibrio* for cell-to-cell signaling. In the past, cell line bioreporters and thin layer chromatography (TLC) have been used to identify AIs in cell media (Pearson et al., 1995, Mion et al., 2021, Zhao et al., 2021, Viswanath et al., 2020). AI atoms were identified with the past method, but they had a low level of affectability, compared to other strategies used in the investigation. With the advancement of advanced technology, for example, high performance liquid chromatography (HPLC), HPLC/mass spectrometry (MS), Gas chromatography (GC)-MS, it is now possible to deliver a comprehensive differentiation proof, and a detailed, unbiased examination of the large number of atoms present in cell culture supernatants (Viswanath et al., 2020).

A few techniques are currently used to illustrate autoinducers and extracellular molecules. By centrifuging bacterial cultures, it is possible to separate the particles from bacterial culture supernatants for characterization. Extraction of AI molecules is predominantly accomplished using liquid–liquid extraction (LLE) and solid phase extraction (SPE). For LLE, an organic solvent is used for the extraction, such as dichloromethane, hexane or acetic acetate. After the extraction, the dissolved substance is dried leaving merely the particles that are activated in methanol (Pearson et al., 1995, Mion et al., 2021).

## 2 Materials and methods

### 2.1 Collection and identification of pathogenic bacteria

To examine cell–cell communication molecules, several types of microscopic organisms belonging to the *Vibrio* genus were used. As a result of the occurrence and prevalence of the bacteria, samples were collected from a variety of sources. Since the aquaculture industry is facing high mortality rates, some pathogenic samples were gathered from this industry. As positive controls, three distinct pathogenic samples were collected from different sources.

We collected moribund shrimps (*Penaeus monodon*) from a shrimp ranch in Nagapattinam, Tamil Nadu. Shrimps were collected in refrigerators and transported to the research facility to be stored at –20 °C (Garrity and Holt, 2001). On sterile filters, infected samples were washed four times with 100 mL of sterile ocean water. Gut and external skin were removed. Homogenization was carried out using a sterile homogeniser and sterile water. We took 25 g of the homogenate and added it to 225 mL of alkaline peptone water (APW) and brooded it for 24 h. Samples were serially diluted up to 10 times. A 100-microliter sample of each was inoculated onto TCBS agar medium. Every plate was incubated between 28 °C and 30 °C. We selected suspected settlements and conducted a variety of biochemical tests.

The other three strains of *V. cholerae*, *V. parahemolytics*, and *V. fischeri* used for the study were obtained from Biotech Research lab, Dept. of Zoology, Thiru. Vi. Ka College, Thiruvarur. The culture was initially activated in LB (Luria Bertanii broth). Approximately one ml of 18 h culture was added to supplement broth over night at 30 °C ± 2 °C in a shaker (Remi, India) at 80 ± 5 rpm. Then, this was reinoculated onto thiosulphate citrate bile sucrose (TCBS) agar for further identification using biochemical responses and based on Bergey's systematic manual of microbiology (Brelles Marino and Eulogio, 2001).

### 2.2 Preparation of cell – Free intracellular supernatants

Using glycerol marine media, the cultures were developed overnight. The following day, the cells were centrifuged at 6000 rpm and resuspended in fresh minimal media. Then, they were centrifuged at 6000 rpm for 10 min. 0.2 µm pore channels were used to filter out bacteria from the supernatant. Three times, the cell-free culture supernatant was extricated with acidified ethyl acetic acetate (99.95% ethyl acetic acid derivation and 0.5% acetic acid utilizing a 1:1 vol proportion). The liquid phase containing the inorganic salts from the media was discarded. The top layer containing the organic phase with the extracellular particles was gathered. To perform GC–MS analysis, the ethyl acetate extricate was dried with N_2_ gas and the dried extracellular media components were disintegrated in CAN water (20%).

### 2.3 Thin layer chromatography (TLC)

The TLC plate (C18 silica) was stacked with sample separators and distinctive principles. Following a few minutes of drying, the plate was then allowed to run in a blend of organic solvents. It was possible to image AHLs isolated with both UV light and chromic agents such as potassium dichromate in sulphuric acid. Further investigation was accomplished by scratching off spots and separating the material with dichloromethane or ethyl acetate for further study (Haque et al., 2021).

### 2.4 High performance liquid chromatography (HPLC)

Concentrates of cell-free concentrates were injected into a HPLC 1200 infinity arrangement, where molecules are pumped by weight through a solid (stationary phase) under a solvent (mobile phase). Molecules collide with and adsorb on the strong material at that point and elute from the segment in different rates. The section measurements were 2.1 × 100 mm and the molecule estimate was 1.8 µm, using octadeconyl carbon chain (C18) - fortified silica for the stationary stage and acetonitrile for the versatile stage. In the segment, non-polar atoms can be partitioned from pH 2–9. We set the draw and discharge speed to 2000 µLmin^−1^, the example volume infused to 5 µL, the suction temperature to 30 °C, and the stream rate to 0.4 µLmin^−1^. The low pressure restraint was set to 0 bars, while the high pressure restraint was set to 900 bars. At one minute, the solvent composition was 95% dissolvable A (water, 5 mM ammonium formate, 0.1% formic acid), and 5% dissolvable B (ACN). After 10 min, the solvent gradient changed to 5% solvent A and 95% solvent B.

### 2.5 Gas chromatography-mass spectrometry (GC–MS)

Here, the sample infusion was done in part mode. Helium was injected at a rate of 1mLmin^−1^. The GC injector was set to 270 °C. After holding 100 °C for a moment, the column oven temperature was expanded at 30°Cmin^−1^ until it reached 300 °C. Mass spectrometry conditions were set at 70 eV, 200 °C, and the solvent cut time was 3.5 min. During the entire sweep, the spectrum was kept in *m*/*z* 200–500 and in SIM mode, at *m*/*z* 143. The samples were measured in SIM mode.

## 3 Results

### 3.1 Collection and identification of isolates

Table 1 shows various isolates of *Vibrio* identified based on their growth in TCBS agar and suspected isolates were subjected to biochemical identification.

**Table 1:** Table showing growth and biochemical characteristics of *Vibrio* on TCBS agar.

Characteristics	SPB1	SPB 2	TC 1	TC 2	TC 3
**Shape**	Short comma	Short comma	Short comma	Short comma	Short comma
**Gram Staining**	-ve	-ve	-ve	-ve	-ve
**Growth on TCBS agar**	Yellow colonies.	Large yellow colonies.	Flat yellow colonies.	Blue to green centered colonies.	Yellow orange colonies.
**Identification**	*V. harveyi*	*V. alginolyticus*	*V. cholerae*	*V. alginolyticus*	*V. fischeri*

### 3.2 Thin layer chromatography (TLC)

**Fig. 1:**
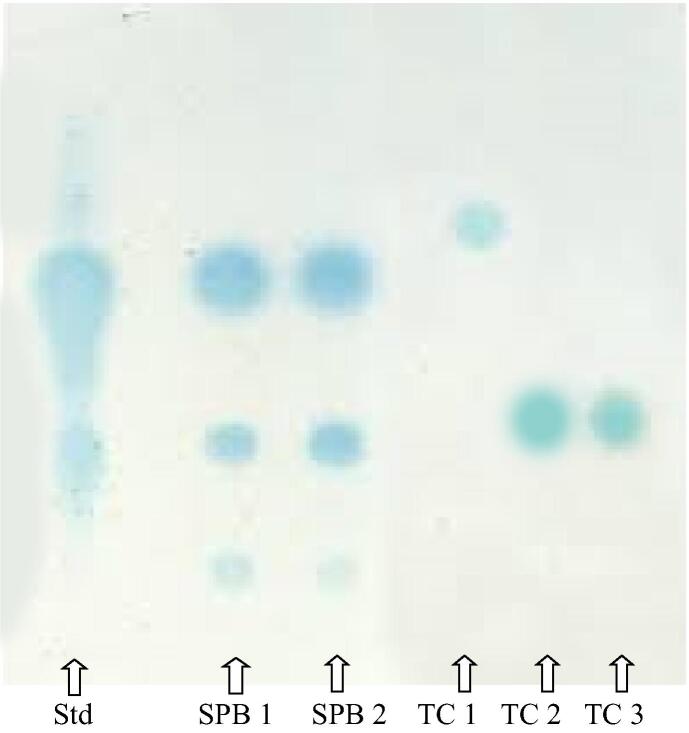
Thin layer chromatogram of various bacterial metabolites.TLC. Std- *Chromobacterium violaceum*, SPB 1-*Vibrio harveyi*, SPB 2-*Vibrio alginolyticus*, TC 1-*Vibrio cholerae*, TC 2-*Vibrio parahemolyticus*, TC 3-*Vibrio fischeri*

Fig. 1 shows the thin layer chromatographic analysis of various bacterial metabolites. Both *V. harveyi* and *V. alginolyticus* showed an exponential rate of AHLs. Additionally, there was a match with the AHL atom delivered by *Chromobacterium violaceum*, a reporter strain. Path 1 represents the acyl homoserine lactone standard detached from *C. violaceum* and alternate paths represent the isolates *V. harveyi*, *V. alginolyticus*, *V. cholerae*, *V. parahemolyticus* and *V. fischeri*. The cellular communication molecules identified by *V. harveyi* and *V. alginolyticus* were only one. The TLC plate has only identified one communication molecule despite the fact that *V. cholerae* contains three communication molecules. Both *V. parahemolyticus* and *V. fischeri* also indicated just one band.

### 3.3 HPLC

**Fig. 2:**
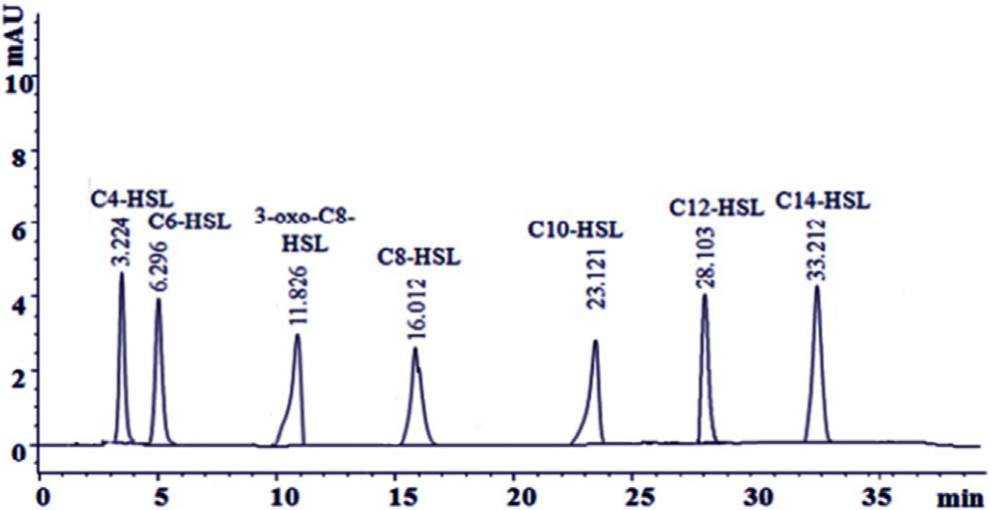
HPLC chromatogram of the predominant AHL producing strain *V. harveyi.*

Fig. 2 shows the HPLC chromatogram of the predominant AHL creating strain *V. harveyi.* Clearly, *Vibrio* produced AHLs with different maintenance times. Add up to 7 tops were shown up with the maintenance times of 3.224 for C_4_-HSL, 6.926 for C_6_-HSL, 11.826 for 3-oxo-C_8_-HSL, 16.012 for C_8_-HSL, 23.121 for C_10_-HSL, 28.103 for C_12_-HSL, and 33.212 for C_14_-HSL.

### 3.4 GC–MS

**Fig. 3:**
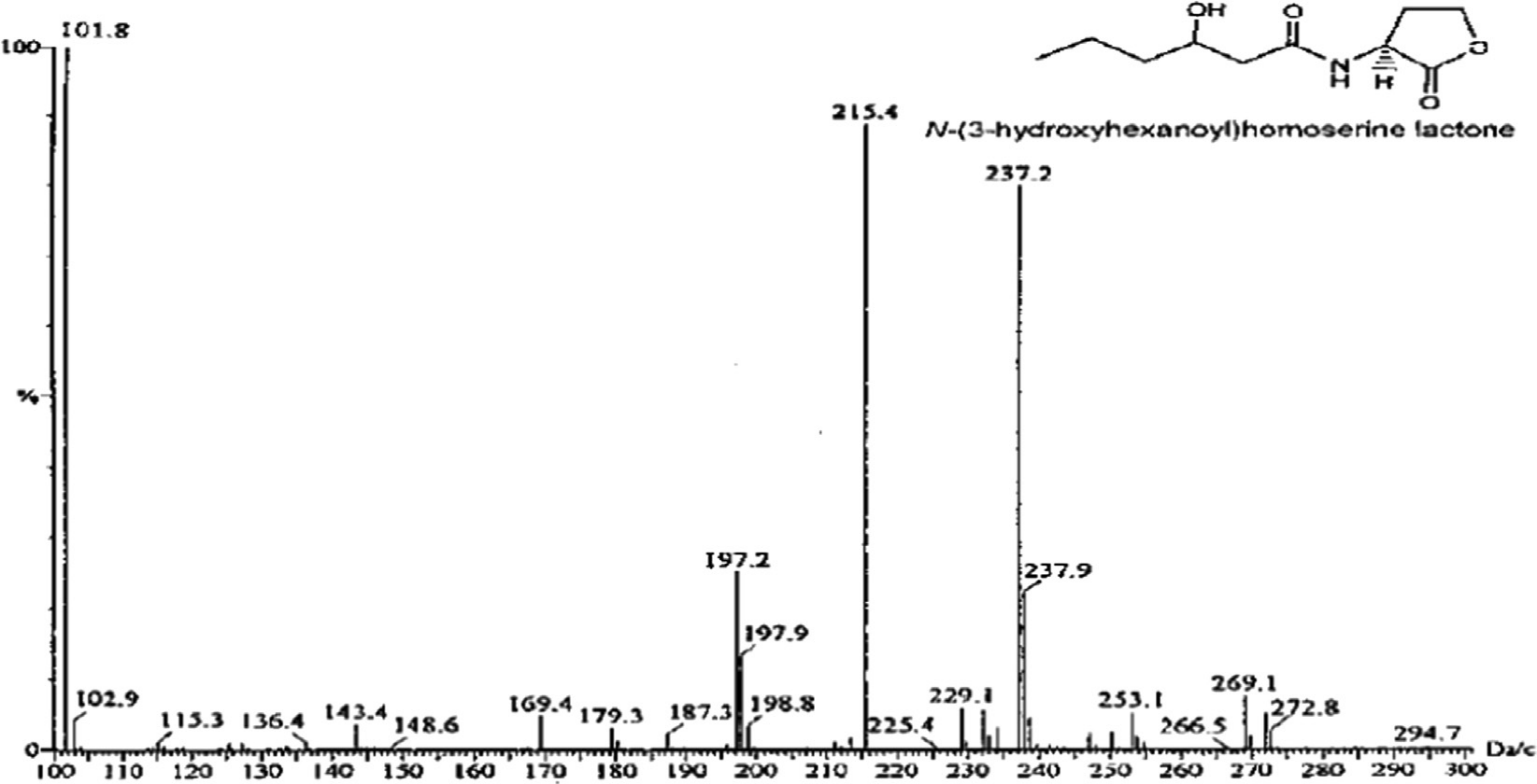
Chromatogram showing different peaks in the mass range of isolated *V. harveyi* strains.

As shown in Fig. 3, different peaks were present in the mass range of isolated *V. harveyi* strains. Among those, the top with maintenance time 215.4 confirmed the existence of AHL atoms. Each compound (crests) also contained the subatomic particle [M] +, the hallmark of homoserine lactone. An abundant fragmentation ion was observed in the AHL.

## 4 Discussion

It is possible to detect AHLs in cell free supernatants using a variety of techniques (13, 14). It is possible to portray AHLs by using TLC on C18 substituted stage plates. The TLC system enables the examination and separation of various AHLs using both natural strategies and expository methods (Viswanath et al., 2020). There are several obstacles to TLC, including the inability to obtain basic data, and the difficulty of maintaining a strategic distance from metabolites (Liu et al., 2017). TLC cannot unambiguously identify AHLs, but their chromatographic properties can provide insight into provisional structures and help differentiate them from standard AHLs. The development of blue spots has been associated with the appearance of AHLs (Calatrava-Morales et al., 2018). Linciano et al. (2020) concluded that TLC is a significant strategy for confirming the generation of AHL atoms (Linciano et al., 2020). According to Acosta-Jurado *et al.* (2020), TLC revealed the occurrence and partition of different AHLs from *Sinorhizobium fredii* HH103 (Acosta-Jurado et al., 2020).

The polar features of the versatile stage cause hydrophobic atoms to adsorb in unison to the stationary stage. Polar mixes have less affinity for reinforced silica and elute first. HPLC analysis shows that the closeness of the 7 tops indicates seven wide range AHLs. In previous studies, specific pinnacles appeared at a maintenance time of 38.123 moments, something that did not take after standard AHL, suggesting the appearance of some unidentified AHL or another metabolite (Acosta-Jurado et al., 2020). Sun *et al.* (2020) described diverse types of AHLs among certain blended cultures (Sun et al., 2020).

Detachment of the HPLC is performed before mass spectrometry. Using HPLC, the sample extracted from LLE is redissolved in methanol, runs through a C18 and C16 reverse phase column, and is eluted with 70% ACN in water or water-methanol (Miller and Gilmore, 2020, Ma et al., 2018). With HPLC, atoms can be partitioned in a practical and simple way with an unusual level of selectivity. A method of ensuring HSL has been developed using mass spectrometry combined with GC. The couple systems of MS indicators provide additional basic information regarding the identity and separation of the signaling particles. Furthermore, GC–MS can be used to detect a majority of particles because of the high chromatographic precision and specificity of the mass indicators. Here, the samples were blended and weakened with acetonitrile to achieve the desired fixation. Helium was used as a transporter gas. Chromatographic information was then collected and recorded in the GC–MS Real Time examination software.

As outlined in the research, GC–MS was used to detect HSLs from gram negative microorganisms (Lee et al., 2021, Stock et al., 2021). An essential objective of GC–MS methodology is to facilitate quick and reproducible data extraction from the sample with a tiny amount of extraction methods. Shin *et al.* (2020) accounted for a comparable mass range for N-butyryl homoserine lactone obtained from *Pseudomonas aeruginosa* (Shin et al., 2019). According to Zhang *et al.* (2021), GC–MS is a suitable method for detecting AHLs, since they are delivered in low concentrations, so that other traditional methods are ineffective (Zhang et al., 2021). Stock *et al.* (2021) found that the AHLs could be resolved at even low fixation levels using GC–MS than the other previously revealed methods (Stock et al., 2021). NCBI's scientific categorization program has classified more than 9000 organisms in the family *Vibrionaceae*. We have considered three prominent QS frameworks in the *Vibrionaceae* family. Lux S/Lux PQ is one of three notable QS frameworks, where Lux S is responsible for the creation of DPD (4,5-dihydroxy-2,3pentanedione), which undergoes an adjustment in proximity to boron in order to produce the auto-inducer particle AI-2 (Zhang et al., 2021). It is widely dispersed in Gram negative and Gram positive microbes. There has been much success with this concept as it has been accepted as the link between species as well as between kingdoms. In prokaryotic and eukaryotic frameworks, methyl transferase catalysts are used to perform a variety of methyl transferase reactions using SAM as the methyl donor (Šimunović et al., 2020, Li et al., 2021, Sela et al., 2021, Zhao et al., 2018). During methylation, SAM becomes SAH, and SAH collected in a cell can be toxic (Barriuso et al., 2018).

A recent report indicated that the *Escherichia coli* Lux S mutant could achieve low levels of AI-2 through the production of ribulose-5-phosphate as a result of glucose fermentation using the oxidative pentose phosphate pathway (Kumaran and Citarasu, 2016). Additionally, two thermo-stable organisms, *Thermotoga sea* and *Pyrococcus furiosus,* delivered AI-2 without Lux S under aqueous conditions (Whiteland et al., 2020). Lux P is the primary collector protein for the AI-2 signal. By studying the communication molecule quorum sensing may be prevented, thus aiding in the fight against diseases in the future.

## 5 Conclusion

Several acyl homoserine lactones have been detected, which are autoinducers in *Vibrio* sp. and confirmed by HPLC and GC–MS. *Vibrio* sp. had seven different AHL molecules associated with their quorum sensing system. By using quorum quenchers, these systems can be blocked, thereby reducing the pathogenicity of *Vibrio* sp. It would be beneficial to study the possibility of using quorum quenchers to reduce the side effects that result from overusing antibiotics by interrupting the communication between *Vibrio* sp.

## Declaration of Competing Interest

The authors declare that they have no known competing financial interests or personal relationships that could have appeared to influence the work reported in this paper.
